# The C/H3 Domain of p300 Is Required to Protect VRK1 and VRK2 from their Downregulation Induced by p53

**DOI:** 10.1371/journal.pone.0002649

**Published:** 2008-07-09

**Authors:** Alberto Valbuena, Sandra Blanco, Francisco M. Vega, Pedro A. Lazo

**Affiliations:** Programa de Oncología Translacional, Instituto de Biología Molecular y Celular del Cáncer, Centro de Investigación del Cáncer, Consejo Superior de Investigaciones Científicas (CSIC)-Universidad de Salamanca, Salamanca, Spain; National Institute on Aging, United States of America

## Abstract

**Background:**

The vaccinia-related kinase 1 (VRK1) protein, an activator of p53, can be proteolytically downregulated by an indirect mechanism, which requires p53-dependent transcription.

**Principal Findings:**

In this work we have biochemically characterized the contribution of several p53 transcriptional cofactors with acetyl transferase activity to the induction of VRK1 downregulation that was used as a functional assay. Downregulation of VRK1 induced by p53 is prevented in a dose dependent manner by either p300 or CBP, but not by PCAF, used as transcriptional co-activators, suggesting that p53 has a different specificity depending on the relative level of these transcriptional cofactors. This inhibition does not require p53 acetylation, since a p53 acetylation mutant also induces VRK1 downregulation. PCAF can not revert the VRK1 protection effect of p300, indicating that these two proteins do not compete for a common factor needed to induce VRK1 downregulation. The protective effect is also induced by the C/H3 domain of p300, a region implicated in binding to several transcription factors and SV40 large T antigen; but the protective effect is lost when a mutant C/H3Del33 is used. The protective effect is a consequence of direct binding of the C/H3 domain to the transactivation domain of p53. A similar downregulatory effect can also be detected with VRK2 protein.

**Conclusions/Significance:**

Specific p53-dependent effects are determined by the availability and ratios of its transcriptional cofactors. Specifically, the downregulation of VRK1/VRK2 protein levels, as a consequence of p53 accumulation, is thus dependent on the levels of the p300/CBP protein available for transcriptional complexes, since in this context this cofactor functions as a repressor of the effect. These observations point to the relevance of knowing the cofactor levels in order to determine one effect or another.

## Introduction

The vaccinia-related kinases (VRK) form a group of three proteins in the human kinome that diverged early from the casein kinase I branch [1]. Several lines of evidence suggest that VRK1 contributes to cell division. Thus, VRK1 is highly expressed in proliferating cell lines [2], and in embryonic development during the expansion of the hematopoietic system [3]. In human biopsies VRK1 is mainly detected in the amplifying compartment of epithelial surfaces, where it co-localizes with several proliferation markers [4]. Loss of human VRK1 by siRNA reduces cell division [5], and in C. Elegans the inactivation of its homolog gene results in embryonic death and arrested growth in adults [6].

The human vaccinia-related kinase 1 VRK1 phosphorylates p53 uniquely in Thr18 [7], [8] and induces its stabilization and acetylation [5]. This specific phosphorylation contributes to p53 stabilization by interfering with binding to hdm2 [5], [9], [10], and increases p53 binding to p300 and p53 acetylation [5]. Differently acetylated p53 molecules may oligomerize with some differences in their organization that can affect gene transcription specificity. The interaction of p53 with hdm2 depends on its phosphorylation. The persistent accumulation of p53 would result in a permanent block to cell cycle progression or the cells will enter apoptosis, and thus is not compatible with life. Therefore p53 levels are usually low and its accumulation is transient. Precisely to prevent this accumulation, p53 induces its main downregulatory protein mdm2/hdm2 [11].

Since VRK1 contributes to p53 stabilization, some mechanism of autoregulation between these two proteins is likely to function in the cell and has been recently identified. In vivo there is an inverse correlation between p53 and VRK1 levels in human tumor cell lines [12]; furthermore in human fibroblast, the induction of DNA damage by ultraviolet light and subsequent accumulation of p53 is accompanied by a downregulation of endogenous VRK1 [12]. This downregulatory mechanism could be reproduced in transfection experiments making it more accessible for characterization [12], and is independent of the promoter used to express VRK1, thus indicating it is an indirect effect [12]. The accumulated p53 regulates VRK1 protein level by proteolytic degradation, which is mediated by an indirect mechanism that requires de novo gene transcription of an unknown gene. The VRK1 downregulation is also independent of a proteasome mediated pathway; this mechanism is insensitive to proteasome inhibitors, and hdm2/mdm2 is not implicated since it is also functional in mdm2 deficient cells [12]. This mechanism targets VRK1 to enter the endosome-lysosome pathway where it is proteolytically downregulated [12]. These autoregulatory properties are altered when p53 is mutated; thus transcription-defective p53 mutants cause an accumulation of VRK1 because its degradation mechanism can not be induced [12], an observation that has been confirmed in human lung squamous cell carcinomas containing mutations in p53, which have very high levels of endogenous VRK1 [13].

Since this VRK1 downregulation requires p53 dependent transcription [12], in this report we have used this VRK1 downregulation by p53 to determine the potential contribution of different acetyl transferase cofactors of p53 that can modulate the specificity of gene transcription, and for which VRK1 downregulation provides a functional assay. The tumor suppressor p53 has different responses to a common stimulation depending on cell type, which is likely to reflect a differential composition of transcriptional complexes. The transcriptional activity of p53 is regulated by interaction with transcriptional coactivators such as the p300/CBP, or the PCAF (p300/CBP-associated factor) acetyl transferases [14]. The sites acetylated in p53 are Lys373, Lys382 by p300/CBP and Lys320 by PCAF [15], which selectively activate transcription [16]. The tumor suppressor p53 interacts with p300 by two different regions, one located in the N-terminus and required for nuclear export and degradation, and the other near the C-terminal region, proximal to but different from the C/H3 region, which is required for activation of transcription [14], [17]. The C/H3 region has an eighty percent homology to the same region in CBP (CREB binding protein), but is not present in PCAF. The C/H3 domain is an interaction region with many different proteins such as viral proteins as adenovirus E1A or SV40 large T antigen, or transcription factors such as MyoD, Fos, c-Jun, and E2F [14], [18].

In this work we have determined the requirement for the contribution of co-transcriptional factors with acetyl transferase activity and determined their contribution to the specificity of the effect induced by p53 on the stability of VRK1. This would indicate that some transcriptional cofactors, but not others will be determinants of the effect. Among the three cofactors, p300, CBP and PCAF that interact with p53, only the former two were able to prevent this VRK1 downregulation. Complexes of p53 with proteins having a C/H3 domain are not able to downregulate VRK proteins.

## Results

### p300 and CBP protect VRK1 from its p53-induced downregulation

**Figure 1:**
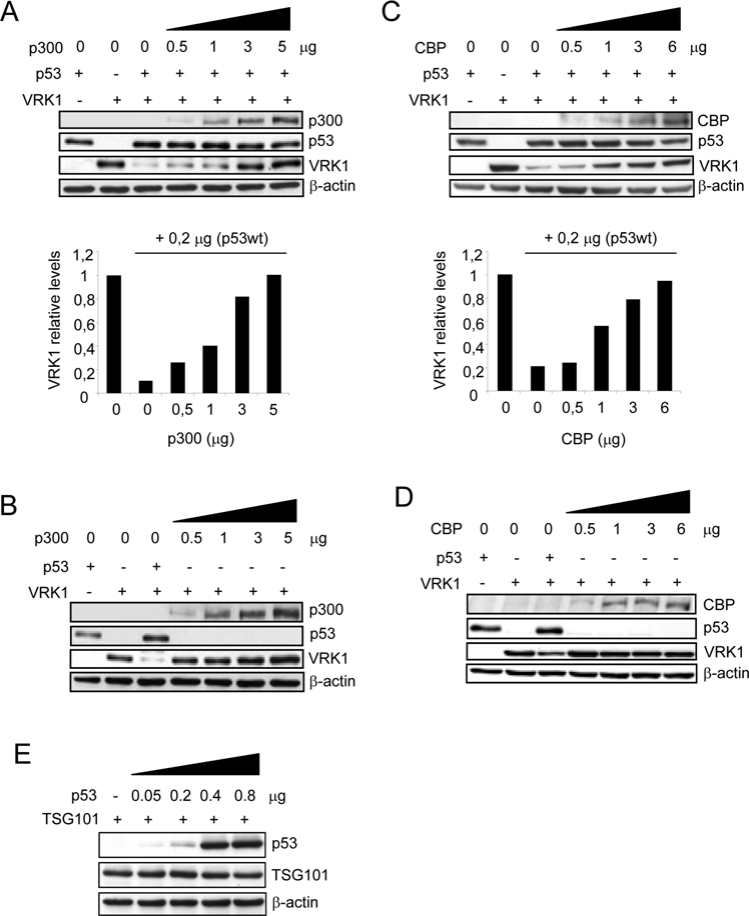
p300 and CBP protect VRK1 from p53-induced downregulation. (A) H1299 cells were transfected with pCB6+p53 (0.2 µg) and pCEFL-HA-VRK1 (5 µg) with increasing amounts of pCMVβ-p300-HA and 36 hours postransfection cell extracts were analyzed with the different antibodies indicated in the legend. Normalized VRK1 protein levels are represented in the graphs. VRK1 and p300 were detected with an anti-HA antibody. (B) p300 over-expression by itself does not have any effect on VRK1 protein levels. An experiment similar to that in part A, but in the absence of p53 was performed. (C) H1299 cells were transfected with pCB6+p53 and pCEFL-HA-VRK1 with increasing amounts of pSG5-CBP and 36 hours postransfection cell extracts were analyzed with the different antibodies indicated in the legend. CBP was detected with a rabbit polyclonal antibody. Normalized VRK1 protein levels are represented in the graphs. (D) CBP over-expression by itself does not have any effect on VRK1 protein levels. An experiment similar to that in part C, but in the absence of p53 was performed. Each protein was detected in the immunoblots as indicated in the methods section. (E). Negative control for lack of p53 effect. H1299 cells were transfected with pCEFL-HA-TSG101 (5 µg) and increasing amounts of pCB6+p53 (0.2 µg). TSG101 was detected with an anti-HA antibody. The experiments were performed independently three times. The quantifications corresponding to immunoblots with differences (parts A–D) are shown and presented in bar graphs.

The downregulation of VRK1 by p53 has been shown to be dependent on p53-induced transcription of an unknown protein that controls VRK1 proteolytic degradation [12]. Therefore it was decided to identify the contribution of p53 transcriptional cofactors to this process. p300 and CBP are p53 coactivators that participate in its transcriptional activation by acetylation of Lys373 and Lys382 residues [14], [19]. It has been previously reported that VRK1 was able to increase p53 acetylation after its specific phosphorylation on Thr-18 [5]. First, it was tested if p300 could have any effect on the transcriptionally dependent downregulation of VRK1 induced by p53. For this aim H1299 cells were cotransfected with plasmids pCEFL-HA-VRK1, pCB6+p53 and increasing amounts of pCMV-p300 (Fig. 1A). Surprisingly, as the p300 protein level increased, it was accompanied by a parallel increase in the protection of VRK1 degradation induced by p53 (Fig. 1A). To confirm that the effect requires the participation of p53, the same experiment was performed in the absence of p53 (Fig. 1B). In this situation p300 over-expression by itself has no effect on VRK1, or perhaps even induces a minor increase in its levels. These results suggested an implication of the p300 coactivator in preventing VRK1 downregulation by p53. An unknown connection between these proteins may occur or it might be possible that the gene controlled by p53 that regulates VRK1 does not require p300 as coactivator and that unique over-expression of p300 is sufficient to direct the transcriptional activity of p53 to other targets and to abrogate the negative effect of p53 on VRK1 levels. The expression of actin was not affected by either p53 or any of the cofactors.

P300 and CBP are two related proteins with an 80 per cent homology suggesting many of their effects are probably similar. Therefore it is likely that CBP could also protect VRK1 from downregulation induced by p53; or alternatively detection of a differential response would contribute to determine the specificity of the effect. To distinguish between these two possibilities, a similar set of experiments was performed. Increasing amounts of CBP were able to protect VRK1 from downregulation induced by p53 (Fig. 1C), and CBP by itself in the absence of p53 had no effect on VRK1 levels (Fig. 1D). As a negative control the lack of effect on another protein that it is not susceptible to this downregulation mechanism was determined. Cells were transfected with a plasmid expressing human TSG101 [20]. The levels of this transfected protein, as well as that of the endogenous actin, are not downregulated by p53 (Fig. 1E).

### PCAF does not protect VRK1 from its p53-induced downregulation

**Figure 2:**
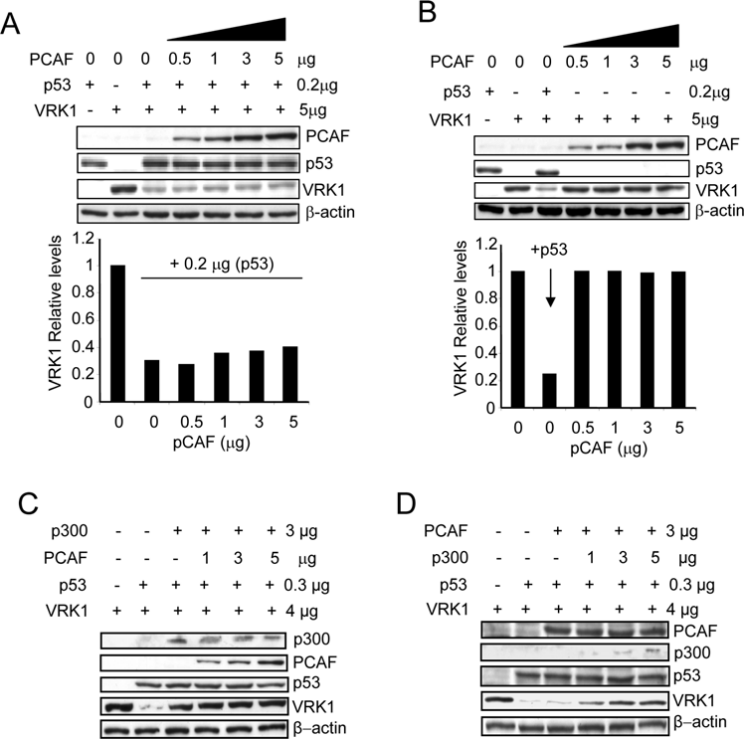
PCAF does not protect from p53-induced downregulation of VRK1. (A) PCAF over-expression does not exert any protective effect on VRK1 downregulation by p53. H1299 cells were transfected with pCB6+p53 (0.2 µg) and pCEFL-HA-VRK1 (5 µg), and the experiment was carried out like in Figure 1, but a PCAF expression plasmid was included instead of the p300 plasmid. VRK1 protein levels were quantified and shown in the graph. PCAF was detected with an anti-Flag antibody. VRK1 was detected with an anti-HA antibody. The quantification of immunoblots is presented in bar graphs. (B) PCAF over-expression by itself, in the absence of p53, does not have any effect on VRK1 protein levels. Normalized VRK1 protein levels are represented in the graph. The quantification of immunoblots is presented in bar graphs. (C). Increasing amounts of PCAF are unable to compete with p300 in its protection of VRK1 downregulation induced by p53. (D). Increasing amounts of p300 protect from p53-induced downregulation of VRK1 independently of the presence of PCAF. The experiments were performed independently three times.

The protein PCAF is another acetyl transferase that also functions as p53 cofactor, and acetylates p53 in a different residue, Lys 320. Therefore, a similar experiment was performed using plasmid pCI-Flag-PCAF, in this case the unique over-expression of the PCAF protein together with p53 and VRK1, in a similar experiment as the one with p300 in the previous section, did not have any protective effect on VRK1 downregulation by p53 (Fig. 2A), and increasing levels of PCAF by itself also had no effect on VRK1 (Fig. 2B). This confirms a specific involvement of p300/CBP protein as a p53 coactivator, which is functionally different form PCAF. It is possible that PCAF might be able to displace p300 from the complex and thus prevent the effect of p300.

To determine if PCAF can compete with p300 in the induction of VRK1 downregulation two types of experiments were performed. First it was tested if increasing amounts of PCAF were able to revert the protection induced by p300, as shown in Fig 2C, PCAF does not revert the effect of p300. Next it was determined if the lack of effect of PCAF could be prevented by p300; increasing amounts of p300 even in the presence of PCAF were able to induce protection of VRK1 (Fig. 2D). Thus it can be concluded that PCAF does not compete with p300 for any factor to induce the response to p53.

### p53 phosphorylation mutants also induce VRK1 downregulation

**Figure 3:**
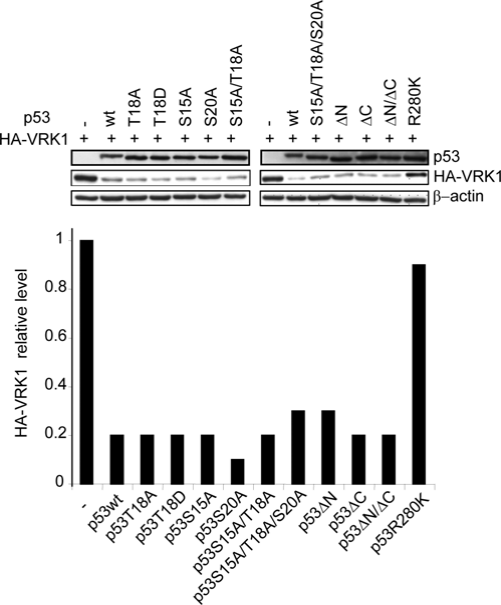
Effect of p53 phosphorylation mutants on the downregulation of VRK1. The p53 aminoacid substitutions are indicated in the diagram. At the top is shown the level of expression of each p53 mutant and at the bottom is shown the quantification of the blot. As positive control is included wild-type p53 that induces the effect (second lane). As negative control for lack of effect the p53^R280K^ mutant is also included. The constructs have mutated either individual, or different combinations of residues as indicated in the legend, or alternatively all p53 phosphorylation residues in the N-terminus (substitutions of phosphorylable residues in the N-terminal region; ΔN: S6A, S9A, S15A, T18A, S20A, S33A and S37A), the C-terminus (substitutions of phosphorylable residues in the C-terminal region; ΔC: S315A, S371A, S376A, S378A, S392A) and both (ΔN/ΔC, all phosphorylatable residues in the N and C-terminal region are substituted). H1299 cells were transfected with 5 µg of pCEFL-HA-VRK1 and the indicated p53 construct. The expression of the p53 constructs aimed to express similar protein levels of all mutants. Cell extracts were prepared 36 hours after transfection and the levels of both proteins were determined by western blot. The transfected VRK1 was detected with an antibody specific for the HA epitope. p53 was detected with a mixture of DO1 and Pab1801 antibodies. The experiments were performed independently three times. The quantification corresponding to the immunoblots shown and is presented in lower bar graphs.

Phosphorylation in the N-terminal region of p53 can modify the interactions of p53 with other proteins and this can, in turn, modify the transcriptional activity of p53 in response to different types of cellular stimulation [21], [22]. However, phosphorylation in this region of p53 appears to be dispensable for the activation of some gene transcription by p53 [23], [24], although it may affect the specificity of interactions with other proteins. Therefore the potential effect of different phosphorylation mutants of p53, either in its amino (ΔN has mutated the following residues: S6A, S9A, S15A, T18A, S20A, S33A and S37A) or carboxy terminus (ΔC has mutated the following residues: S315A, S371A, S376A, S378A, S392A) [25], were tested to determine if they have any effect on the p53 induction of VRK1 protein downregulation. Several p53 mutants affecting individual or all phosphorylatable residues were also tested. All the p53 phosphorylation mutants, including p53^T18D^ that mimics phosphorylation by VRK1 and prevents interaction with hdm2 [12], induced a VRK1 downregulation like the wild-type p53 (Fig. 3). In this experiment, a p53 mutant that is transcriptionally inactive by mutation in the DNA binding domain, p53^R280K^, was included as a positive control for loss of effect, and as expected [12] it did not induce a VRK1 downregulation (Fig. 3).

### p300 prevents VRK1 downregulation induced by the p53^L22Q/W23S^ mutant

**Figure 4:**
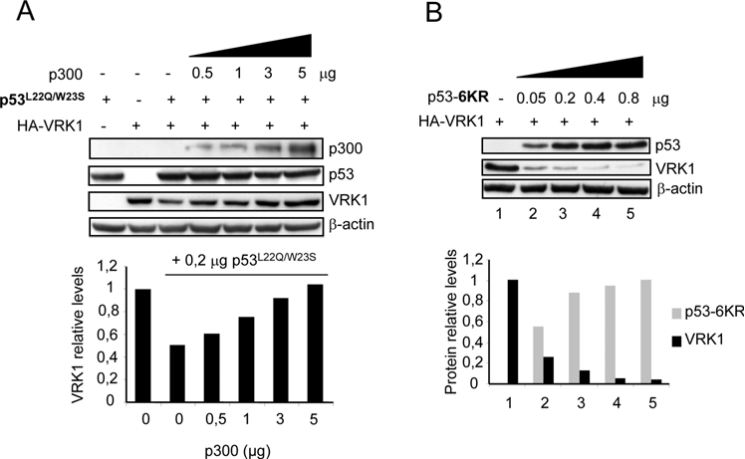
Protection of VRK1 downregulation by p300 is independent the p53 transactivation domain (A) and of p53 acetylation (B). (A) H1299 cells were transfected with the indicated amounts of the p53^L22Q/W23S^ conformational mutant that prevents binding to acetyl transferases as p300, 5 µg of pCEFL-HA-VRK1 and increasing amounts of p300. VRK1 was detected with an anti-HA antibody. (B). H1299 cells were transfected with the indicated amounts of the p53-6KR acetylation mutant and 5 µg of pCEFL-HA-VRK1. Cell extracts were prepared 36 hours after transfection and the levels of both proteins were determined by western blot. To the bottom is shown the quantification of the blots to illustrate the changes in both proteins. The transfected VRK1 was detected with an antibody specific for the HA epitope. The experiments were performed independently three times. The quantification corresponds to the immunoblots shown and is presented in bar graphs.

The transactivation domain of p53 interacts with p300, and this interaction is partially disrupted in the p53^L22Q/W23S^ conformational mutation [17], which has been shown to bind weakly to p300, thus it is defective as a gene repressor and in apoptosis induction [26]; but this mutant is still able to downregulate VRK1 [12]. Therefore it was tested if this downregulatory effect could be prevented by p300, even if the direct interaction of p53–p300 is impaired. For this aim H1299 cells were transfected with the p53^L22Q/W23S^ plasmid and pCEFL-HA-VRK1 in the absence or presence of p300 (Fig 4A). In this case p300 still protected VRK1, although much less efficiently, from the downregulation induced by p53^L22Q/W23S^ (Fig 4A). This observation is consistent with its reduced interaction with p300. But this p53^L22Q/W23S^ mutant is very inefficient in inducing VRK1 downregulation [12], thus there is very little margin to detect a protection by p300.

### Non acetylated p53 also induces downregulation of VRK1

The three proteins p300, CBP and PCAF are histone acetylases, therefore one possibility is that their differential effect might be mediated by acetylation of p53 in different residues. For this purpose it was determined if the use of a p53 mutant, which has all its six acetylated lysine residues in its oligomerization C-terminal domain mutated, was still able to induce VRK1 downregulation. This p53-6KR equally induced downregulation of VRK1 indicating that acetylation was not necessary, and that the effect must be due to specific interactions in the p300 molecule (Fig. 4B). Therefore it can be concluded that p53 induced downregulation of VRK1 is not associated with the acetylation of p53.

### The C/H3 region of p300 can block the p53-induced downregulation of VRK1

**Figure 5:**
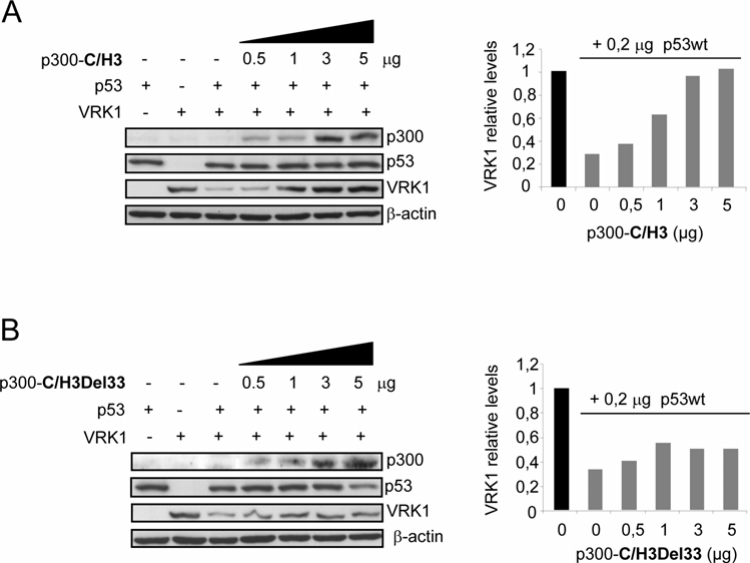
Effect of the p300 C/H3 domain. (A) Effect of the complete p300 CH3 domain that lacks acetyl transferase activity. H1299 cells were transfected with the indicated amounts of the p300-C/H3-Flag plasmid, pCB6+p53 (0.2 µg) and 5 µg of pCEFL-HA-VRK1. VRK1 was detected with an anti-HA antibody. P300 was detected with an anti-Flag antibody. (B) Effect of the p300 C/H3Del33 domain lacking the residues required for interaction with SV40 large T antigen. H1299 cells were transfected with the indicated amounts of the p300-C/H3-Del33-Flag plasmid pCB6+p53 (0.2 µg), and 5 µg of pCEFL-HA-VRK1. The CH3 domains were detected with an anti-Flag antibody. The experiments were independently performed three times. The quantification corresponds to the immunoblots shown and is presented in bar graphs.

The p300 molecule has two different regions of interaction with other molecules. One of them is located near its N-terminal region and is associated with p53 degradation, and requires participation of hdm2/mdm2. The second p300 region of protein interactions is more proximal to the C-terminus and is implicated in activation of transcription. The C/H3 binding domain of p300, which has an 86 per cent homology with CBP, is a region implicated in their interactions with SV40 large T antigen, adenovirus E1A, PCAF, Fos, E2F and c-Jun proteins [27]. The p300 HAT activity is located in residues 1195 to 1921, but is lost if residues 1353–1355 and 1466–1467 are mutated or deleted [17]. Therefore two different constructs expressing this p300 binding domain, p300-C/H3-Flag (aa 1709–1913) and p300-C/H3-Del33-Flag (same region but lacking aminoacids 1737–1809 required for binding to the SV40 large T antigen) [17] do not have acetyl transferase activity. Thus any effect will be a consequence of their protein interaction and not of the enzymatic activity. Expression of the C/H3 construct by itself was able to prevent the induction of VRK1 degradation by p53 (Fig. 5A), but this prevention was lost if the deletion C/H3-Del33 defective in binding was used (Fig. 5B). These results indicate that the possible interaction of p53 with the C/H3 region (aminoacids 1709–1913) of p300 is enough to change its transcriptional specificity, or compete for a common factor shared with p53, and thus modifies the effects induced by a p53 dependent mechanism. The effect is lost by deletion (Del33) (aminoacids 1709–1913 without residues 1737–1836) of the interaction region with SV40 large T antigen, a viral polymerase, suggesting that the mechanism might be controlling the specificity of the interaction of p300 with cellular polymerases and their association or integration in transcriptional complexes.

**Figure 6:**
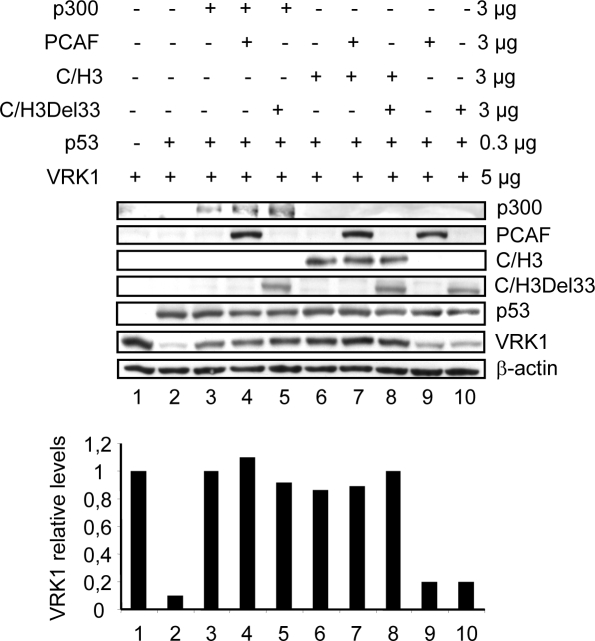
PCAF or C/H3Del33 do not compete with p300 or C/H3 in the protection of VRK1. H1299 cells were transfected with the indicated amount of plasmids in different combinations. p300 was detected with an antibody anti-p300 at 1∶1000 dilution. PCAF, p300-C/H3 and p300-C/H3Del33 were detected with an anti-flag antibody.

Next it was determined if either PCAF or C/H3-Del33 could compete in the protective effect mediated by either p300 of the C/H3 domain (Fig. 6). The effect is shown in lanes 1 to 3, but neither PCAF (lane 4) nor C/H3Del33 (line5) could revert the protection mediated by p300. Next the protective effect of C/H3 (lane 6) was not affected by either PCAF (lane 7) or C/H3Del33 (lane 8). The last two lanes (9 and 10) are controls to show the lack of protection of PCAF or C/H3Del33 by themselves.

### The p300 C/H3 domain directly binds to the transactivation domain of p53

**Figure 7:**
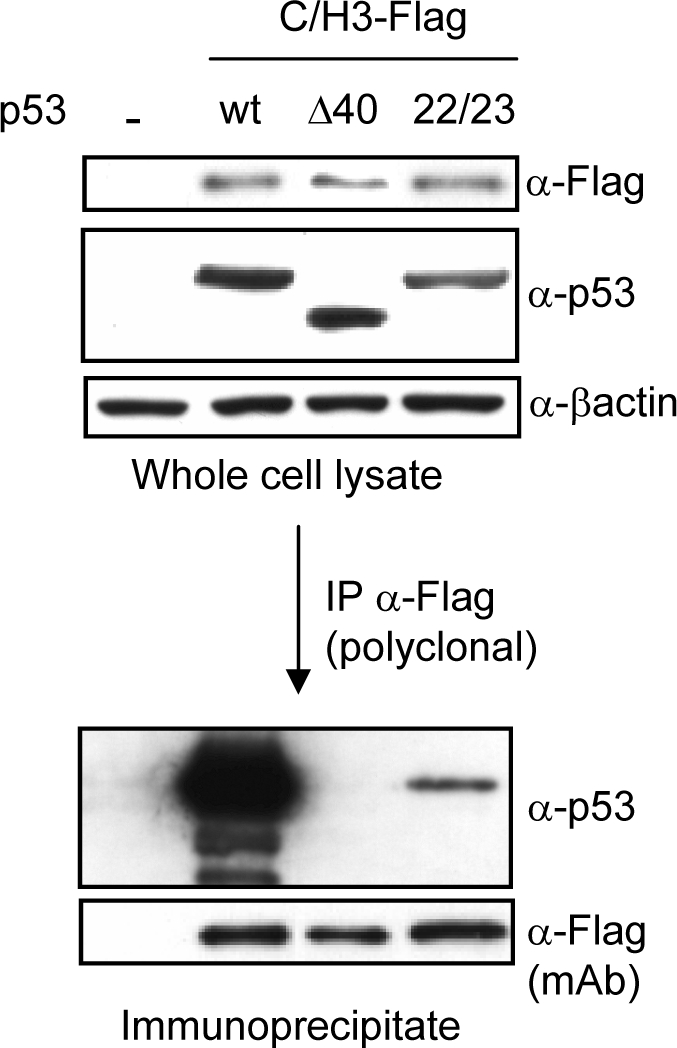
p300 C/H3 domain directly binds to transactivation domain of p53. H1299 cells were transfected with plasmids expressing wild-type p53, pCB6+p53 (0,6 µg), its isoform lacking the transactivation domain pCMV-Δ40p53 (5 µg) or the conformational mutant pCMV-p53^L22Q/W23S^ (0,6 µg), in combination with plasmids p300-C/H3-Flag (5 µg). At the top is shown the level of expression of each construct in whole cell lysates that were used for immunoprecipitation. The Flag tagged proteins were immunoprecipitated with an anti Flag polyclonal antibody, and the p53 bound was determined in an immunoblot using anti p53 specific antibody (bottom gel). In the immunoprecipitate C/H3 was detected with an anti-Flag monoclonal antibody.

A major difference between p300, CBP and PCAF is that the first two proteins have a C/H3 domain. Although the role of C/H3 as a binding region for p53 is not clear, and the evidence is contradictory [14], [17], [18], [28], C/H3 is located in within p300 C-terminal region required for interaction with the SV40 large T antigen and several transcription factors [17]. Therefore, it is possible that the mechanism by which p300/CBP, or for that matter their C/H3 domain blocks VRK1 downregulation might be precisely because the C/H3 region directly interacts with p53 and thus prevents its effect. To test this possibility H1299 cells were transfected with different constructs of wild-type p53, the conformational mutant p53^L22Q/W23S^, or Δ40p53 isoform lacking the first 40 amino acids of the transactivation domain [12], [29]; and their interaction with the C/H3 domain was determined in immunoprecipitation experiments. The C/H3 domain interacted with the wild-type p53, and this interaction was lost if it lacks the transactivation domain, while the interaction was much weaker with the conformational mutant p53^L22Q/W23S^ (Fig. 7), consistent with its defective role as gene repressor [26], but retains other functions in DNA repair [30]. These data are consistent with the reduced VRK1 protection shown in previous experiments (Fig. 4). These results suggest that binding of proteins by their C/H3 domain to the transactivation domain of p53 are likely to affect the type of transcriptional complexes formed, and thus the specificity of gene activation; implying that the one required to induce VRK1 downregulation is not activated when proteins with a C/H3 domain form part of the complex. The binding of the C/H3 to p53 may be functioning as a dominant negative factor.

### VRK2 isoforms are also downregulated by p53 and the effect is prevented by p300 or chloroquine

**Figure 8:**
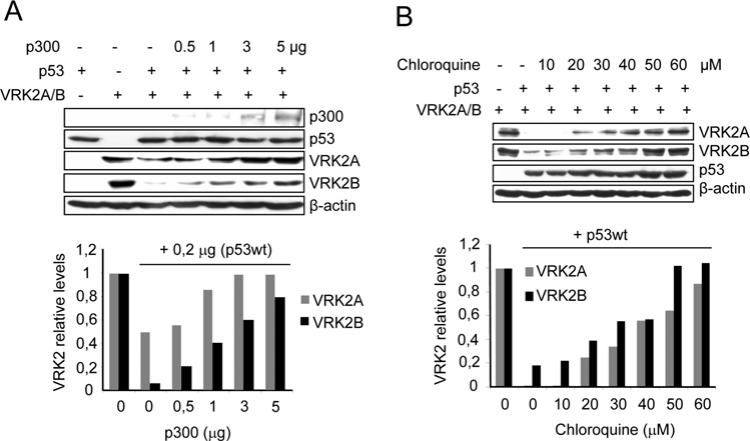
p53 also induces downregulation of VRK2 isoforms that is protected by p300 and is sensitive to chloroquine. (A). Effect of p300 on VRK2A or VRK2B downregulation. H1299 cells were transfected with pCB6+p53 (0.2 µg) and 5 µg of pCEFL-HA-VRK2A or pCEFL-HA-VRK2B plasmids. p300 was expressed from increasing amounts (µg) of plasmid. VRK2 and p300 were detected with an anti-HA antibody. The quantification of normalized levels of VRK2A and VRK2B are shown at the bottom. (B). Effect of chloroquine, an inhibitor of the endosome-lysosome vesicle transport. Cells were transfected with plasmids as indicated. The quantification of normalized levels of VRK2A and VRK2B are shown at the bottom.

The human VRK2 gene generates by alternative splicing two isoforms of VRK2 of 508 (VRK2A) and 397 (VRK2B) aminoacids respectively [31]. The two isoforms are identical in their first 396 aminoacids, thus VK2B is a variant that lacks the C-terminal domain, which contains the membrane anchor of VRK2A [31], [32]. Both VRK2 isoforms have the conserved endosomal-lysosomal target sequence, therefore it is highly likely that they should also be downregulated by the same mechanism as VRK1 [12]. Furthermore VRK2 also phosphorylates p53 in the same residue as VRK1 [31]. Therefore it was tested if the two VRK2 isoforms, A (bound to the endoplasmic reticulum) and B (mostly nuclear), were also downregulated by high levels of p53. H1299 cells were transfected with each of the VRK2 isoforms and high levels of p53. The amount of p53 that induced downregulation of VRK1 was also able to downregulate both isoforms of VRK2 (first two lanes in Fig. 8A). In order to determine if the underlying mechanism was the same, two experiments were performed. First it was established if the level of p300 was also able to protect VRK2 isoforms from downregulation. Cells were transfected with increasing amounts of p300 in the presence of p53 at a level that downregulated VRK2 isoforms. P300 was able to protect both VRK2 isoforms from p53-induced downregulation in a p300 dose dependent manner (Fig. 8A)

Next it was determined if VRK2 downregulation was also sensitive to inhibitors of endosome-lysosome vesicular transport, such as chloroquine. For this experiment cells were transfected with a fixed amount of p53 that induces an almost complete downregulation of both VRK2 isoforms, and the effect of an increasing concentration of chloroquine was determined. Chloroquine inhibited in a dose-dependent manner the downregulation of VRK2A and VRK2B induced by p53 (Fig. 8B). Therefore it was concluded that VRK2A and B, like VRK1, protein levels were down regulated by a similar mechanism that requires p53 and that is sensitive to the level of p300, and to inhibitors of the endosome-lysosome pathway.

### VRK1 and VRK2 have multiple endocytic-lysosomal regions

**Figure 9:**
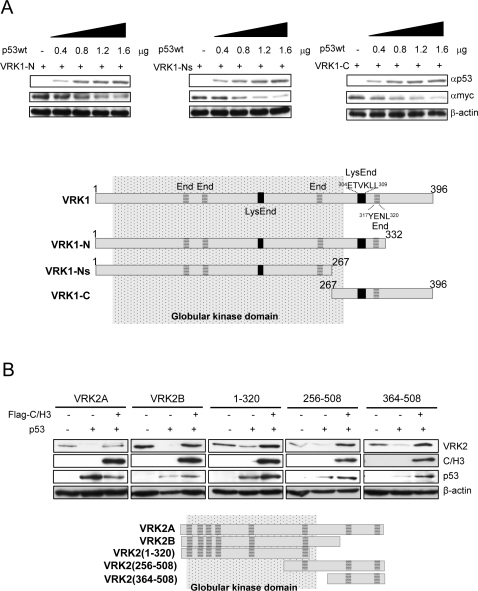
Mapping in VRK1 and VRK2 the regions needed for degradation. (A). Three deletion constructs of human VRK1 were tested for their sensitivity to induction of degradation by p53. Linear sequence motifs were identified using the ELM program (Eukaryotic Linear Motif program) available from the European Molecular Biology Laboratory. End. Sequence for endosomal target motif (grey horizontal boxes); LysEnd: sequence for lysosomal-endosomal target motif (black boxes). The VRK1 truncation constructs were detected with an antibody recognizing the myc epitope used as tag. (B). Identification of target sequences in VRK2 to enter the degradation pathway induced by p53 and its protection conferred by the C/H3 domain of p300. VRK2 was detected with an anti-HA antibody. The C/H3 domain of p300 was detected with an anti-Flag antibody.

VRK1 has a region located between residues 304–320 that has a consensus sequence for targeting VRK1 to the lysosomal-endosomal pathway, which was shown to be the route of VRK1 proteolytic degradation induced by p53 [12]. Several VRK1 constructs spanning different regions of VRK1 were tested for their downregulation induced by p53. All VRK1 deletion mutants were downregulated (Fig. 9A). These results suggested that more than one target sequence is likely to exist in the VRK1 protein. VRK1 has an accessible endocytic and lysosomal target sequences in region 304–320. However, other sequences for endosomal targeting that are not normally exposed, but may be available in the deletion proteins because probably they do not fold correctly the globular kinase domain. VRK1 protein has several potential regions for endocytic-lysosomal targeting (Fig. 9A). Additional targeting sequences that can mediate interaction with the endosomal-adaptor protein are located in positions 107–110, 126–129, 191–196, and 249–252, but they are not exposed because they are embedded within the globular kinase domain; however some may be exposed if in deletion constructs the protein folding is not correct (Fig. 9A). Thus all deletion constructs have more than one sequence that targets them to enter the endocytic pathway if exposed, and this may explain why all of them are sensitive to this downregulation.

Similarly different constructs of the human VRK2 proteins were also downregulated by p53 and protected by the C/H3 domain of p300 (Fig. 9B). These data indicate that there are at least two regions in the protein with sequences that can target VRK2 proteins for degradation. Analysis with ELM programs identifies one of them in residues 422–425, but VRK2 has several additional sequences than might bind the mu subunit of the adaptor protein that may be exposed in VRK2 deletion constructs. These latter sequences are evenly distributed throughout the sequence at positions 44–47, 77–80, 116–119, 238–241 and 306–309 (Fig. 9B).

## Discussion

The direct mechanism implicated in the downregulation of VRK1 protein is mediated by a p53-dependent gene, since different p53 mutants, including the most common mutations detected in human cancers, which are not able to induce transcription does not cause downregulation of VRK1 protein [12]. A mechanism that was confirmed in human squamous cell lung carcinomas, where those cases harboring p53 mutations presented very high levels of VRK1 protein [33]. Furthermore, as shown in this work analysis of p53 phosphorylation mutants in residues implicated in responses to stress, which are not essential for transcription [23], [24], have a similar effect to that of wild-type p53 on VRK1 downregulation. This mechanism induces the targeting of VRK proteins to enter in a pathway that result in their proteolytic downregulation; but the mechanism by which VRK1, or VRK2 proteins are targeted is not yet known. This downregulation of VRK1, or VRK2, induced by p53 requires transcriptional activation of an intermediate gene that is responsible for targeting VRK proteins to enter the endocytic-lysosomal pathway [12], as shown by its sensitivity to chloroquine. VRK1 plays an early role in cell cycle progression [34]; and VRK2 regulates signal transduction in response to hypoxia [32] or interleukin-1β [35] by interaction with components of MAP kinase pathways.

In this work we have analyzed the contribution of acetyl transferases cofactors to regulate the induction by p53 of this VRK1 downregulatory effect. The relevance of p53 transcriptional specificity is manifested by the observation that overexpression of p300 or CBP can block the effect of p53, but the PCAF coactivator does not. This might be due to the promoter specificity of different p53-transcriptional cofactor complexes [36]. But PCAF and p300 do not compete for the same cofactor; since the p300 protective effect is detected even in the presence of excess PCAF. This p300 inhibitory competition of VRK1 downregulation is independent of acetyl transferase activity, since it is blocked by proteins that do not have the acetyl transferase activity as is the case for the C/H3 region of p300. Also the downregulatory mechanism does not require p53 acetylation since it is also induced by a p53 protein containing all its acetylation sites replaced. The inhibitory mechanism can be explained by a competition for binding to p53 by proteins containing a C/H3 domain, such as p300/CBP. Other acetyl transferase such as PCAF, lacking a C/H3 domain, has no effect. The C/H3 domain depletes the p53 molecules needed for induction of VRK1 downregulation. The C/H3 domain of p300/CBP proteins is an interaction region known to bind to several transcription factors such as MyoD, Fos, c-Jun, or E2F and to viral proteins such as adenovirus E1A or SV40 large antigen [18], and also to the transactivation domain of p53. Functionally, these interactions have been shown to have competing effects, thus C/H3 mediates a stimulation of c-Fos that is blocked by binding to E1A [37]. The inhibitory effect of p300/CBP, or their isolated C/H3 domain, is the consequence of a successful competition for p53, because of its direct interaction, which is also required for the transcriptional complex of p53 needed to induce the indirect downregulation of VRK1. This competition effect is lost in the presence of the C/H3Del33 defective domain, or by PCAF that does not have a C/H3 region. Mechanistically these observations are consistent with a dominant negative role for the C/H3 domain [38]–[40].

The interaction of p53 with these cofactors is affected by the residue phosphorylated [41]; for example, Ser15 phosphorylation, or its aspartic substitution, favors binding to p300; while its substitution by alanine results in a much weaker interaction [42]. The association with these cofactors in transcriptional complexes is necessary for specific gene expression. Differential protein associations determine p300 and CBP specificity [43] in processes such as in myogenesis [44]; p300/CBP modulates the BRCA1 inhibition of estrogen receptor [45], and downregulation of p300/CBP activates a senescence checkpoint in melanocytes [46]. PCAF, but not p300/CBP, acetylates the transcription factor Fetal-Kruppel-like factor (FKLF2) [47]. PCAF acetylates PTEN and reduces its ability to down-regulate phosphatidylinositol 3-kinase signaling and to induce G1 cell cycle arrest [48]. But also p300 and PCAF can cooperate in activation in Notch responses [49]; and both p300/CBP and PCAF can acetylate p53 in response to DNA damage [50]. Also CBP and PCAF can acetylate MyoD increasing its transcriptional activity [51]. Differential effects of p53 cofactors have also been reported in the apoptotic response that requires p300 but not CBP [52] in response to ionizing radiation [53]. In the context of cancer, it is important to note that, in the presence of activated H-Ras or N-Ras oncogenes, an active degradation of p300 is induced [54], and this change will permit the activation and subsequent degradation of VRK1 by the new complexes of p53. Among the genes regulated by p53 there is a clear candidate to be implicated in this process. The expression of *DRAM* is positively regulated by p53, and encodes a lysosomal protein implicated in degradation of stable proteins [55], as is the case of VRK1 [34]. VRK1 degradation is promoted by DRAM (unpublished results). Inducible active degradation of stable proteins is an important step in biological processes such as autophagy [55].

Target selection by transcriptional complexes, in which proteins such as p53 or transcriptional cofactors with acetyl transferase activity are implicated, are very likely to be affected by the relative intracellular concentrations of these proteins; and depending on their intracellular concentrations, one or another group of genes may be stimulated. However, the role of relative changes in factor concentration has so far received little attention, in comparison with all or none effects, despite their very important physiological relevance. The effect of changing levels of p300/CBP on the induction of VRK1 proteolytic downregulation, requiring p53 dependent transcription as an intermediate step, can be considered in this context of factor competition. Thus the downregulation of VRK1 is not only determined by the level of p53, but VRK1 downregulation is also conditioned by the relative levels of different acetyl transferases present in the cell. It is highly likely that the heterogeneity of effects frequently observed in response to common types of stimulation is precisely reflecting differences in the intracellular balance of the proteins implicated.

## Materials and Methods

### Plasmids, antibodies and reagents

The VRK1 construct, pCEFL-HA-VRK1, coding for the wild type VRK1 has been previously described [5]. Similarly pCEFL-HA-VRK2 (A and B) has been reported [31]. The N-terminal region of VRK1 (residues 1–267), lacking the exposed endocytic targeting region was cloned in pCDNA3.1 as a *Kpn*I-*Xho*I fragment. The plasmid pCB6+p53 and its different phosphorylation mutants [25] and the acetylation mutant p53-6KR were from Dr. K. Vousden (The Beatson Institute, Glasgow); plasmid pCMVβ-p300-CHA was from Richard Eckner (University of Zurich, Switzerland); and plasmids p300-C/H3-Flag and p300-C/H3Del33-Flag were from J. DeCaprio (Harvard University, Boston, MA) [17]; plasmid pCI-FLAG-PCAF was from Y. Nakatani (Dana Farber Cancer Institute, Boston). pSG5-CBP was from D. Heery (Nottingham University, UK). Plasmid pCEFL-HA-TSG101 was made by subcloning human TSG101 cDNA in vector pCEFL-HA (S. Blanco, unpublished). All plasmids used for transfection were endotoxin free and purified with the JetStar Maxi kit from Genomed (Bad Oeynhausen, Germany).

VRK1 was detected using a rabbit polyclonal antibody (VE1), or a mouse monoclonal antibody (1F6 clone), made against a VRK1 fusion protein [13]. VRK2 isoforms were detected with a specific polyclonal antibody [31]. A mouse monoclonal antibody HA-probe (F7) against the HA tag was from Covance (Berkeley). The p53 protein was detected with a mixture of DO1 antibody (Santa Cruz, CA) and Pab1801 (Santa Cruz, CA) used at 1∶500 and 1∶1000 respectively. p300 was detected with RW128 monoclonal antibody (Upstate, Lake Placid, NY). CBP was detected with sc-583 rabbit polyclonal antibody (Santa Cruz). The anti β-actin (AC-15) antibody was from Sigma. As secondary antibody a Goat anti-mouse-HRP and Goat anti-rabbit-HRP (Amersham Pharmacia Biotech) were used at 1∶5000 in immunoblots.

### Cell lines and transfections

The human lung cancer cell line H1299 (p53^−/−^) was grown in RPMI supplemented with 10% fetal calf serum, glutamine, penicillin and streptomycin in a humidified atmosphere and 5% CO_2_. For transfection experiments H1299 cells were plated in 60 or 100 mm dishes and transfected with the plasmid indicated in the specific experiments with JetPEI reagent following manufacturer's instructions (Polytransfection, Illkirch, France). The cells were, unless otherwise indicated, lysed 36 hours postransfection, in lysis buffer (Tris-HCl 50 mM pH 8, 150 mM NaCl, 5 mM EDTA and 1%Triton X-100 plus protease and phosphatase inhibitors) and 25 µg of whole cell extract were processed for SDS-PAGE and subject to immunoblotting with the indicated antibodies. Where indicated, chloroquine, an inhibitor of endosome-lysosome fusion was added at the indicated concentration twelve hours after transfection and cells were lysed after an additional twenty-four hours.

### Immunoprecipitations

Whole cell extracts (1 mg) were precleared by incubation with 50 µl of Gamma-Bind G Sepharose beads (GE Healthcare) for 30 min at 4°C and washed 3 times in lysis buffer (10 mM EDTA, 10% Glycerol, 140 mM NaCl, 1% NP40, 20 mM TrisHCl pH 8.0). The corresponding antibody was added to the cleared cell extract and incubated overnight with rotation at 4°C. Next 40 µl of Gamma-Bind G Sepharose beads blocked with PBS and 1% BSA were added and incubated for 4 hours at 4°C. The beads were washed five times in lysis buffer[13]. The washed beads were used for loading gels and immunoblot analysis.

### Immunoblots

Total protein extracts were quantified using a BIORAD Protein assay kit (Biorad, Hercules, CA). Proteins were fractionated in an SDS-polyacrylamide gel and transferred to a PVDF Immobilon-P membrane (Millipore). The membrane was blocked with TBS-T buffer (25 mM Tris, 50 mM NaCl, 2.5 mM KCl, 0.1% Tween-20) and 5% defatted-milk. Afterwards the filter was rinsed with TBS-T buffer and the specific primary antibody (indicated in individual experiments) added and incubated for 90 minutes at room temperature. The filter was rinsed and incubated with a secondary antibody conjugated with peroxidase for 30 minutes. The membrane was develop for chemiluminescence with the ECL reagent (Amersham, Little Chalfont, UK) and exposed to X-ray films (Fuji).

To detect high molecular proteins such as p300 or CBP, a 5% PAGE was run at low voltage for 5 hours, running out of the gel lower molecular size proteins up to 250 kDa. The proteins were transferred in buffer (25 mM TrisHCl, 192 mM glycine) with 10% methanol overnight at 4°C. The detection of p300 and CBP were detected with the indicated antibodies. All experiments were performed three times and all immunoblots were quantified in the linear response range; however in figures is shown a representative western blot and its quantification.
